# Endonasal Surgery after Cocaine Abuse: Safe at Any Interval?

**DOI:** 10.1155/2012/823470

**Published:** 2012-07-09

**Authors:** L. K. Døsen, R. Haye

**Affiliations:** The Department of Otorhinolaryngology, Lovisenberg Diakonale Hospital, 0440 Oslo, Norway

## Abstract

*Objective*. We report a case of poor healing after endonasal surgery for nasal septal perforation ten years after cocaine abuse was ended. *Method*. The clinical findings are presented. *Results*. A 35-year-old man presented with a small nasal septal perforation caused by cocaine abuse. He had stopped using it ten years previously so surgery was considered safe. The perforation was surgically closed using an endonasal approach. The perforation, however, recurred, the incision healing delayed, and a saddle deformity developed. *Conclusion*. The effects of cocaine abuse seem to persist causing poor healing after nasal surgery. Prosthetic treatment should be the primary choice. Caution should be employed when considering surgery even in small perforations due to cocaine abuse even many years after the abuse was terminated.

## 1. Introduction

Cocaine is used for its topical vasoconstrictive and anesthetic effect. It is also a powerful psychostimulant. Repeated nasal insufflation causes local ischemia leading to tissue destruction and even septal nasal perforation. Surgeons therefore are reluctant to perform nasal surgery on current or even former drug abusers [1]. It has, however, been suggested that nasal surgery could be safe if there has been no abuse for one or more years [2–4] providing there is no relapse of the abuse. We present this case to warn against endonasal surgery even when the patient has been off cocaine for many years.

## 2. Case Report

A nonsmoking, healthy, 35-year-old man complained of nasal stuffiness, crusting, and bleeding due to a nasal septal perforation. This had been caused by nasal insufflation of cocaine for one year ten years previously. He initially used only one dose (25 mg) weekly but this dose was taken five times the last week before the perforation occurred. Eight weeks later cautery with silver nitrate was applied once unilaterally to a bleeding point at the posterior edge of the perforation. He had been off the cocaine abuse for ten years at the time of surgery. This is confirmed by his employment as an officer in the merchant marine during these years. There was no history of nasal trauma, surgery, or long-term use of nasal sprays. The perforation measured 5 × 5 mm. There was crusting at the posterior end. No other nasal abnormality was observed, the mucoperichondrium looked healthy and the cartilage seemed strong at palpation. The surgical closure was performed by an experienced surgeon using an endonasal approach. The incision started as a hemitransfixion on the right side, continued along the nasal sill to the inferior concha and thereafter dorsally underneath the concha until the equivalent position of the posterior end of the perforation and then curved medially to the middle of the nasal floor. A lower mucoperiosteal/perichondrial flap was then created and the nasopalatine artery and nerve severed so the flap could be brought medially and upwards along the nasal septum to close the perforation. The same procedure without the hemitransfixion incision was performed on the left side. The mucoperichondrium above the perforation was left in place. A piece of cartilage and bone was then brought forwards into the perforation to obtain a three layered closure. The perforation and the incisions were carefully adapted with PDS sutures making sure the blood supply was not compromised. Silicone sheaths were placed endonasally for protection and slight compression. They were removed after nine days revealing a defect at the site of the perforation. The hemitransfixion incision had failed to close. Oral and topical antibiotics were given despite absence of infection. A reperforation occurred after two weeks and the hemitransfixion incision widened. The incision closed after a further two weeks; a cartilaginous saddle deformity was then apparent. The size of the perforation (3 × 3 mm) has remained unchanged one year later.

## 3. Discussion

Endonasal surgery for nasal septal perforation is usually a safe procedure with very good results and few complications [5–8]. Cartilaginous saddle deformity can occasionally be seen if the upper mucoperichondrial flaps have been created bilaterally and moved inferiorly leaving the upper part of the cartilage without blood supply [8–10]. Delayed closure of the hemitransfixion incision, however, has not been described. In the present case we experienced three different complications that all are related to poor healing. The cause of these might be due to complications during surgery. However, there were no abnormal events, surgical mistakes, infection, tight packing, or tight sutures. We believe, therefore, that there must be an additional reason for the failure.

During endonasal surgery as described, the blood supply to the anterior part of the nose (branches from the superior labial and nasopalatine arteries) is reduced. The lower flaps are nearly entirely dependent on the blood supply from the sphenopalatine arteries via the posterior nasal arteries. However, surgery performed in this manner has never caused all three complications in our hands.

Unilateral cautery is not associated with widespread tissue destruction, delayed healing of incisions, or cartilage destruction (saddle deformity). The cautery took place ten years before the surgery and so it is unlikely that the cautery has had any effect upon the surgical healing.

Cocaine has an immediate vasoconstrictive effect. Biopsies have shown that it also causes pseudovasculitis with inflammatory cells encroaching on the wall of the vessels causing narrowing of the lumen [1, 11, 12]. Thrombosis and sclerotic changes in the vascular wall representing postthrombotic scarring have also been described [1]. Little, however, is known of the long-term effects of these events. We believe that the prior use of cocaine must have played a part in the surgical failure by creating a permanently less viable tissue or vascular insufficiency.

This suggests that caution should be employed when considering surgery of nasal septal perforation due to cocain abuse even many years after the abuse was terminated. We recommend that nasal silicone buttons should be considered as the primary choice of treatment of nasal septal perforation caused by cocaine. The long-term results of prosthetic treatment, however, are less favorable than surgery [13–15].
